# Disparities in Preoperative Goals of Care Documentation in Veterans

**DOI:** 10.1001/jamanetworkopen.2023.48235

**Published:** 2023-12-19

**Authors:** Adela Wu, Karleen F. Giannitrapani, Ariadna Garcia, Selen Bozkurt, Derek Boothroyd, Alyce S. Adams, Kyung Mi Kim, Shiqi Zhang, Matthew D. McCaa, Arden M. Morris, Scott Shreve, Karl A. Lorenz

**Affiliations:** 1 VA Health Services Research and Development Center for Innovation to Implementation, VA Palo Alto Health Care System, U.S. Department of Veterans Affairs, Palo Alto, California; 2 Department of Neurosurgery, Stanford University School of Medicine, Stanford, California; 3 Department of Primary Care and Population Health, Stanford University School of Medicine, Stanford, California; 4 Quantitative Sciences Unit, School of Medicine, Stanford University, Stanford, California; 5 Evaluation Sciences Unit, School of Medicine, Stanford University, Stanford, California; 6 Department of Epidemiology and Population Health, Stanford University, Stanford, California; 7 Office of Research Patient Care Services, Stanford Health Care, Palo Alto, California; 8 S-SPIRE Center, Department of Surgery, School of Medicine, Stanford University, Palo Alto, California; 9 Veterans Affairs Palo Alto Health Care System, US Department of Veterans Affairs, Palo Alto, California; 10 Lebanon VA Medical Center, US Department of Veterans Affairs, Lebanon, Pennsylvania; 11 Penn State College of Medicine, Hershey, Pennsylvania

## Abstract

**Importance:**

Preoperative goals of care discussion and documentation are important for patients undergoing surgery, a major health care stressor that incurs risk.

**Objective:**

To assess the association of race, ethnicity, and other factors, including history of mental health disability, with disparities in preoperative goals of care documentation among veterans.

**Design, Setting, and Participants:**

This retrospective cross-sectional study assessed data from the Veterans Healthcare Administration (VHA) of 229 737 veterans who underwent surgical procedures between January 1, 2017, and October 18, 2022.

**Exposures:**

Patient-level (ie, race, ethnicity, medical comorbidities, history of mental health comorbidity) and system-level (ie, facility complexity level) factors.

**Main Outcomes and Measures:**

Preoperative life-sustaining treatment (LST) note documentation or no LST note documentation within 30 days prior to or on day of surgery. The standardized mean differences were calculated to assess the magnitude of differences between groups. Odds ratios (ORs) and 95% CIs were estimated with logistic regression.

**Results:**

In this study, 13 408 patients (5.8%) completed preoperative LST from 229 737 VHA patients (209 123 [91.0%] male; 20 614 [9.0%] female; mean [SD] age, 65.5 [11.9] years) who received surgery. Compared with patients who did complete preoperative LST, patients tended to complete preoperative documentation less often if they were female (19 914 [9.2%] vs 700 [5.2%]), Black individuals (42 571 [19.7%] vs 2416 [18.0%]), Hispanic individuals (11 793 [5.5%] vs 631 [4.7%]), or from rural areas (75 637 [35.0%] vs 4273 [31.9%]); had a history of mental health disability (65 974 [30.5%] vs 4053 [30.2%]); or were seen at lowest-complexity (ie, level 3) facilities (7849 [3.6%] vs 78 [0.6%]). Over time, despite the COVID-19 pandemic, patients undergoing surgical procedures completed preoperative LST increasingly more often. Covariate-adjusted estimates of preoperative LST completion demonstrated that patients of racial or ethnic minority background (Black patients: OR, 0.79; 95% CI, 0.77-0.80; *P* <.001; patients selecting other race: OR, 0.78; 95% CI, 0.74-0.81; *P* <.001; Hispanic patients: OR, 0.78; 95% CI, 0.76-0.81; *P* <.001) and patients from rural regions (OR, 0.91; 95% CI, 0.90-0.93; *P* <.001) had lower likelihoods of completing LST compared with patients who were White or non-Hispanic and patients from urban areas. Patients with any mental health disability history also had lower likelihood of completing preoperative LST than those without a history (OR, 0.93; 95% CI, 0.92-0.94; *P* = .001).

**Conclusions and Relevance:**

In this cross-sectional study, disparities in documentation rates within a VHA cohort persisted based on race, ethnicity, rurality of patient residence, history of mental health disability, and access to high-volume, high-complexity facilities.

## Key Points

**Question:**

What factors, including race, ethnicity, rurality of residence, and Veterans Affairs (VA) facility complexity level, are associated with disparities in veterans completing preoperative life-sustaining treatment (LST) documentation?

**Findings:**

In this cross-sectional study of 13 408 patients, few patients undergoing surgical procedures completed preoperative LST, with disparities in documentation rates based on race, ethnicity, rurality of patient residence, history of mental health disability, and access to high-volume facilities within a VA cohort.

**Meaning:**

These findings suggest that there is continued need for interventions that target patient groups at risk, namely racial or ethnic minority groups and those with history of mental health conditions, of missing opportunities to engage in serious illness communication.

## Teaser

This cross-sectional study assesses the association of race, ethnicity, and other factors, including history of mental health disability, with disparities in preoperative goals of care documentation among a population of patients of the Veterans Healthcare Administration.

## Introduction

Surgery is a significant health care occurrence that should prompt timely goals of care (GOC) planning and discussions about current personal values, goals, and treatment preferences. Best practices guidelines from the American College of Surgeons and the American Geriatrics Society recommend GOC discussion and documentation by health care clinicians, including surgeons, in case patients may not be able to make their own medical decisions.^1^ However, rates of GOC discussion and documentation are poor, and only 6.1% of preoperative consultations included the discussion of treatment preferences and goals for those undergoing high risk surgery, despite surgeons upholding the importance of preoperative GOC planning.^2^

Moreover, inadequate GOC documentation serves to amplify existing health care disparities. Patients identifying as Hispanic, Asian, and Black have been found to be significantly less likely to have discussions with their health care clinicians and to have documented directives.^3,4^ A observational study focused on patients older than 65 years in California also demonstrated that male patients and those who spoke non-English preferred languages were significantly less likely to complete GOC discussion and documentation.^5^

From 2017, the Veterans Health Administration (VHA) has piloted and coordinated the Life-Sustaining Treatment (LST) Decisions Initiative (LSTDI) to encourage GOC discussion and documentation. The LSTDI is an effort to engage health care clinicians and patients in discussing and documenting patients’ wishes and preferences regarding various medical treatments for prolonging life and designation of surrogate decision makers. Since the national implementation of the LSTDI in July 2018 until February 2020, only 3.8% of VHA patients undergoing surgical procedures had completed LST documentation by the time of their surgery despite recommendations to conduct the LST process for seriously ill patients at risk for life-threatening events.^6^

This study was informed by the conceptual framework of access to health care from Levesque et al^7^ as well as published literature on health care services delivery within the VHA system.^8^ Thus, we performed a cross-sectional study and selected known or hypothesized factors associated with disparities in GOC discussion and LST documentation for VHA patients undergoing surgical procedures. While the LSTDI had relatively low implementation rates at the outset, we aimed to study populations at risk or characteristics associated with differences in preoperative access and documentation of GOC.^6^ Thus, the goals for our investigation were to (1) investigate potential disparities in preoperative LST documentation based on patient’s racial or ethnic background and health conditions; (2) identify patient-level and system-level associations with preoperative LST documentation; and (3) describe the COVID-19 pandemic’s association with the completion of preoperative LST. We hypothesized that patients undergoing surgical procedures from minority backgrounds and patients with vulnerabilities, such as a history of mental health disability, would be at risk of missing opportunities to engage in preoperative GOC planning and LST documentation.

## Methods

The cross-sectional study was approved by the joint Veterans Affairs (VA) Palo Alto Healthcare System and Stanford University institutional review board (IRB). A waiver of study participant consent was obtained from the IRB committee because patient data were deidentified and publicly available and the study was deemed minimal risk. The study followed the Strengthening the Reporting of Observational Studies in Epidemiology (STROBE) reporting guideline for cross-sectional studies. We included all veterans who underwent at least 1 surgical procedure between January 1, 2017, and October 18, 2022, in the VHA and were using VHA services for at least 1 year prior to their surgical procedure.

### Data Source

To construct our cohort, we obtained data from the VA Corporate Data Warehouse (CDW). Patients who had missing variables were excluded from the final analysis. Missing variables excluded 7% of the original cohort. We did not impute race if that value was missing.

**Figure 1.**
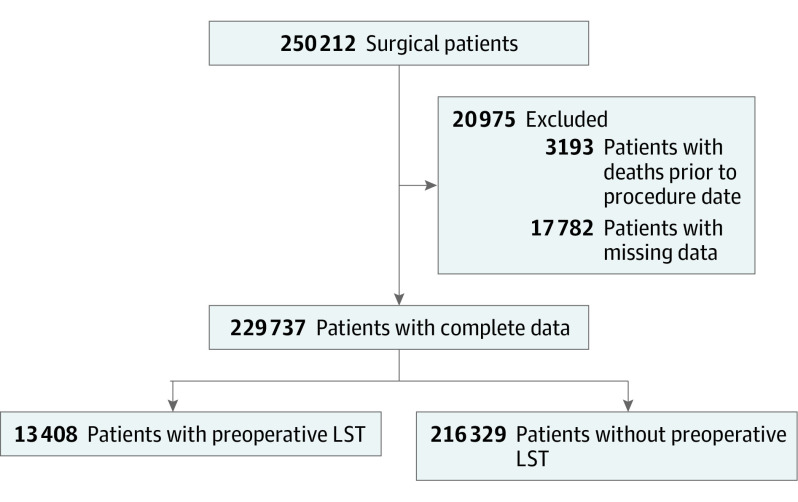
Flowchart of Cohort LST indicates life-sustaining treatment.

Using categories created from clinical evaluation of *International Statistical Classification of Diseases, Tenth Revision, Clinical Modification *(*ICD-10-CM*) codes and classified by Healthcare Cost and Utilization Project (HCUP) procedure class flags, we included surgical procedures that were defined as major therapeutic or diagnostic procedures performed in the operating room for our analysis.^9^ Further designation of surgical procedures as high risk or non-high risk was based on prior work and *ICD-10-CM* codes.^6,10^ Figure 1 illustrates the cohort creation.

### Outcomes

Our primary outcome was preoperative LST documentation within 30 days prior to surgery.^11^ We searched the CDW for a health factor metric named *ethics-life-sustaining treatment*, which indicated that an LST progress note had been activated. LST documentation was defined by completion and documentation of at least 4 mandatory elements: GOC about values and overall treatment purpose, resuscitation preferences, decision-making capacity or designation of a surrogate, and consent.^12^ Partial completion or no completion of these elements was considered incomplete LST documentation.^12^ Other LST components could include preferences regarding artificial nutrition and hydration.

### Patient- and System-Level Characteristics

Our study was informed by the conceptual framework of access to health care and the health equity implementation framework.^7,13^ Race as a social construct is a widely accepted concept in multiple fields, including anthropology and sociology. The VHA collects patient information about race as a singular reported entity (American Indian, Asian, Black, Hispanic Black, Hispanic White, White, and unknown), reported either by self or by proxy. Importantly, veterans who identify as multiracial or multiethnic may not be reflected accurately in the CDW. We categorized race into 3 groups: Black or African American, White, and other (including American Indian or Alaskan Native, Asian, Native Hawaiian, or Other Pacific Islander). We defined ethnicity as Hispanic vs non-Hispanic individuals.

We also examined the association between LST completion and several other patient characteristics, including age, sex (male or female), marital status (married; divorced, widowed, or separated; and single or never married), housing instability, rurality of patient residence (rural vs urban), Care Assessment Need score within 1 year prior to day of surgery, Charlson Comorbidity Index (CCI) within 1 year prior to day of surgery, diagnoses of serious medical comorbidities (end stage kidney disease, cancer, cardiopulmonary arrest, dementia, frailty), history of mental health comorbidity (major depression, posttraumatic stress disorder, psychotic disorders, bipolar disorder), and substance use disorder (the use of 1 or more of the following substances: alcohol, methamphetamine, cocaine, opiates, and sedative or anxiolytic) based on *ICD-10-CM* codes within the year prior to surgery.^6,12^

To investigate the potential moderating effect of rurality with the association of patient race or ethnicity and preoperative LST completion, we included an interaction term between these variables in our prespecified analysis.^12^ Surgical specialty types (cardiothoracic surgery, general surgery, neurosurgery, orthopedic surgery, urology, vascular surgery, and other), surgical risk level (high risk or nonhigh risk), VA facility complexity, which is a reflection of patient volumes and resources for clinical, research, and teaching on site, and procedure year were used to account for temporal trends in the adjusted analysis.^11,14^ Table 1 indicates the definitions of VA facility levels.

**Table 1.** Veterans Affairs Facility Complexity Level Definitions^a^

Level	Complexity	Definition
1A	High	High volume, high-risk patients, most complex clinical programs, and large research and teaching programs
1B	High	Medium-high volume, high-risk patients, many complex clinical programs, and medium-large research and teaching programs
1C	Medium	Medium-high volume, medium-risk patients, some complex clinical programs, and medium-sized research and teaching programs
2	Medium	Medium volume, low-risk patients, few complex clinical programs, and small or no research and teaching programs
3	Low	Low volume, low-risk patients, few or no complex clinical programs, and small or no research and teaching programs

^a^ Data are from the National Academies of Sciences, Engineering, and Medicine.^14^

### Statistical Analysis

We summarized patient-level and system-level characteristics descriptively based on preoperative LST completion status. We calculated standardized mean differences (SMDs) to assess the magnitude of differences between the groups. We defined an SMD value of 0.2 as the threshold for determining meaningful differences between the groups.^15^ SMDs were calculated using the R package tableone version 0.13.2 (R Project for Statistical Computing).^16^ Our approach treated a multinomial variable as multiple nonredundant dichotomous variables and used the Mahalanobis distance, a measure between a sample point and a distribution, to calculate SMDs.^17^ Categorical variables were summarized as percentages, with the frequency and percentage of each category reported. Continuous variables were reported as mean (SD).

We used logistic regression to model the association between LST completion and patient characteristics, while adjusting for the complexity level of the VA facility where the surgery took place to estimate the odds ratios (ORs) and 95% CIs for each variable in the model. We included a patient’s VA facility type as a fixed effect to account for clustering. To account for potential correlations within clusters of patients, we used a sandwich estimator to obtain robust standard errors and calculate 95% CIs.^18^ To account for potential temporal confounding, including the onset of the COVID-19 pandemic, we adjusted for procedure year in the statistical model. To assess the potential collinearity of the variables, we examined the Pearson correlation coefficients among independent variables. We used the variance inflation factor (VIF) to assess multicollinearity among explanatory variables and found that all VIF values were less than 5, indicating no issues with multicollinearity. We performed a sensitivity analysis on the logistic regression results by repeating the main analyses on an expanded cohort with completed LST documentation within 90 days before surgery.

All statistical analyses were performed using R version 4.0.5 (R Project for Statistical Computing) between October 2022 and February 2023. All *P* values were calculated based on 2-sided tests, and statistical significance was determined at the threshold of *P* < .05.

## Results

Of the 229 737 veterans (209 123 [91%] male; 20 614 [9.0%] female; mean [SD] age, 65.5 [11.9] years) at 130 VA facilities included in this study, 13 408 (5.8%) completed an LST note within 30 days prior to surgery (Table 2). While SMD values varied, there were differences in patients who did and did not complete a documented preoperative LST. In unadjusted analyses, the patients who did not complete LST preoperatively compared with those who completed preoperative LST documentation tended to be younger (18 to 54 years: 32 802 [15.2%] vs 1092 [8.1%]; 55 to 64 years: 52 419 [24.2%] vs 2558 [19.1%]), female (19 914 [9.2%] vs 700 [5.2%]), Black individuals (42 571 [19.7%] vs 2416 [18.0%]), Hispanic (11 793 [5.5%] vs 631 [4.7%]), resided in rural areas (75 638 [35.0%] vs 4273 [31.9%]), had no history of substances used (185 788 [85.9%] vs 11 103 [82.8%]), had a history of 1 or more mental health comorbidities (65 974 [30.5%] vs 4053 [30.2%]), had no history of housing instability (200 833 [92.8%] vs 12 280 [91.6%]), and were seen at low-complexity, low-volume facilities designated as level 3 (7849 [3.6%] vs 78 [0.6%]). Patients presenting to highest-complexity facilities designated as level 1A (7215 [53.8%] vs 114 704 [53.0%]) and level 1B (3462 [25.8%] vs 48 340 [22.3%]) completed preoperative LST more frequently. Throughout the study, unadjusted rates of preoperative LST documentation continued to increase (173 [0.27%] in 2017; 2917 [4.3%] in 2018; 4009 [7.0%] in 2019; 2733 [12.6%] in 2020; 2091 [16.7%] in 2021; and 1485 [18.3%] in over three quarters of 2022). Patients who underwent surgery after March 11, 2020, the date when the World Health Organization declared the COVID-19 pandemic, tended to complete preoperative LST documentation more frequently (5484 [40.9%] vs 28 679 [13.3%]).^19^

**Table 2.** Cohort Baseline Characteristics

Characteristics	Overall	Patients, No. (%)		SMD
		Did not complete preoperative LST	Completed preoperative LST	
Total	229 737	216 329 (94.2)	13 408 (5.8)	
Age group, y				
18-54	33 894 (14.8)	32 802 (15.2)	1092 (8.1)	0.33
55-64	54 977 (23.9)	52 419 (24.2)	2558 (19.1)	
65-84	13 2476 (57.7)	123 853 (57.3)	8623 (64.3)	
≥85	8390 (3.7)	7255 (3.4)	1135 (8.5)	
Age, mean (SD), y	65.5 (11.9)	65.3 (11.86)	69.7 (11.1)	0.38
Sex				
Female	20 614 (9.0)	19 914 (9.2)	700 (5.2)	0.15
Male	209 123 (91.0)	196 415 (90.8)	12 708 (94.8)	
Race				
Black	44 987 (19.6)	42 571 (19.7)	2416 (18.0)	0.06
White	5717 (2.5)	168 304 (77.8)	10 729 (80.0)	
Other^a^	179 033 (77.9)	5454 (2.5)	263 (2.0)	
Ethnicity				
Hispanic	12 424 (5.4)	11 793 (5.5)	631 (4.7)	0.03
Non-Hispanic	217 313 (94.6)	204 536 (94.5)	12 777 (95.3)	
Marital status				
Married	113 172 (49.3)	107 057 (49.5)	6115 (45.6)	0.08
Divorced, widowed, or separated	89 642 (39.0)	83 930 (38.8)	5712 (42.6)	
Single or never married	26 923 (11.7)	25 342 (11.7)	1581 (11.8)	
Rurality				
Rural	75 393 (32.8)	75 637 (35.0)	4273 (31.9)	0.07
Urban	149 827 (65.2)	140 692 (65.0)	9135 (68.1)	
CAN score (1 y)				
<80	199 472 (86.8)	190 993 (88.3)	8479 (63.2)	0.61
≥80	3277 (1.4)	2909 (1.3)	368 (2.7)	
Not available	26 988 (11.7)	22 427 (10.4)	4561 (34.0)	
CAN score, mean (SD)	24.9 (19.7)	24.6 (19.4)	33.35 (22.8)	0.42
Charlson Comorbidity Index				
0 (Lowest comorbidity level)	56 995 (24.8)	55 203 (25.5)	1792 (13.4)	0.44
1-3	100 148 (43.6)	95 229 (44.0)	4919 (36.7)	
≥4 (High comorbidity level)	72 594 (31.6)	65 897 (30.5)	6697 (49.9)	
Comorbid disease				
ESKD	9123 (4.0)	8194 (3.8)	929 (6.9)	0.40
Cardiopulmonary disease	46 619 (20.3)	42 830 (19.8)	3789 (28.3)	
Cancer	59 259 (25.9)	55 630 (25.7)	3629 (27.1)	
Dementia	539 (0.2)	472 (0.2)	67 (0.5)	
Frailty	29 674 (12.9)	27 564 (12.7)	2110 (15.7)	
Other	37 610 (16.4)	36 047 (16.7)	1563 (11.7)	
Not available	46 913 (20.4)	45 592 (21.1)	1321 (9.9)	
Substance use				
No	196 891 (85.7)	185 788 (85.9)	11 103 (82.8)	0.09
Yes^b^	32 846 (14.3)	30 541 (14.1)	2305 (17.2)	
Mental Health Comorbidity				
No	159 710 (69.5)	150 355 (69.5)	9355 (69.8)	0.01
Yes^c^	70 027 (30.5)	65 974 (30.5)	4053 (30.2)	
Housing Instability				
No	213 113 (92.8)	200 833 (92.8)	12 280 (91.6)	0.05
Yes	16 624 (7.2)	15 496 (7.2)	1128 (8.4)	
Surgical risk				
Not high-risk	174 485 (75.9)	163 932 (75.8)	10 553 (78.7)	0.07
High-risk	55 252 (24.1)	52 397 (24.2)	2855 (21.3)	
Surgical subspecialty				
General	57 931 (25.2)	54 495 (25.2)	3436 (25.6)	0.28
Neurosurgery	21 040 (9.2)	20 194 (9.3)	846 (6.3)	
Cardiothoracic	35 621 (15.5)	33 037 (15.3)	2584 (19.3)	
Vascular	15 515 (6.8)	14 610 (6.8)	905 (6.7)	
Orthopedic	77 239 (33.6)	72 157 (33.4)	5082 (37.9)	
Urology	13 036 (5.7)	12 767 (5.9)	269 (2.0)	
Other	9355 (4.1)	9069 (4.2)	286 (2.1)	
Procedure year				
2017	63 132 (27.5)	62 959 (29.1)	173 (1.3)	1.03
2018	67 388 (29.3)	64 471 (29.8)	2917 (21.8)	
2019	56 932 (24.8)	52 923 (24.5)	4009 (29.9)	
2020	21 643 (9.4)	18 910 (8.7)	2733 (20.4)	
2021	12 523 (5.5)	10 432 (4.8)	2091 (15.6)	
2022	8119 (3.5)	6634 (3.1)	1485 (11.1)	
COVID-19 pandemic	34 163 (14.9)	28 679 (13.3)	5484 (40.9)	0.66
Prior to COVID-19 pandemic	195 574 (85.1)	187 650 (86.7)	7924 (59.1)	
VA facility complexity				
1A	121 919 (53.1)	114 704 (53.0)	7215 (53.8)	0.23
1B	51 802 (22.5)	48 340 (22.3)	3462 (25.8)	
1C	34 428 (15.0)	32 654 (15.1)	1774 (13.2)	
2	13 661 (5.9)	12 782 (5.9)	879 (6.6)	
3	7927 (3.5)	7849 (3.6)	78 (0.6)	

Abbreviations: CAN, Care Assessment Need score; ESKD, end-stage kidney disease; LST, life-sustaining treatment; SMD, standardized mean difference; VA, Veterans Affairs.

^a^ Other race includes American Indian or Alaskan Native, Asian, Native Hawaiian, or Other Pacific Islander.

^b^ One or more of the following: alcohol, methamphetamine, cocaine, opiate, sedative or anxiolytic.

^c^ One or more of the following: major depression, posttraumatic stress disorder, psychotic disorders, bipolar disorder.

**Figure 2.**
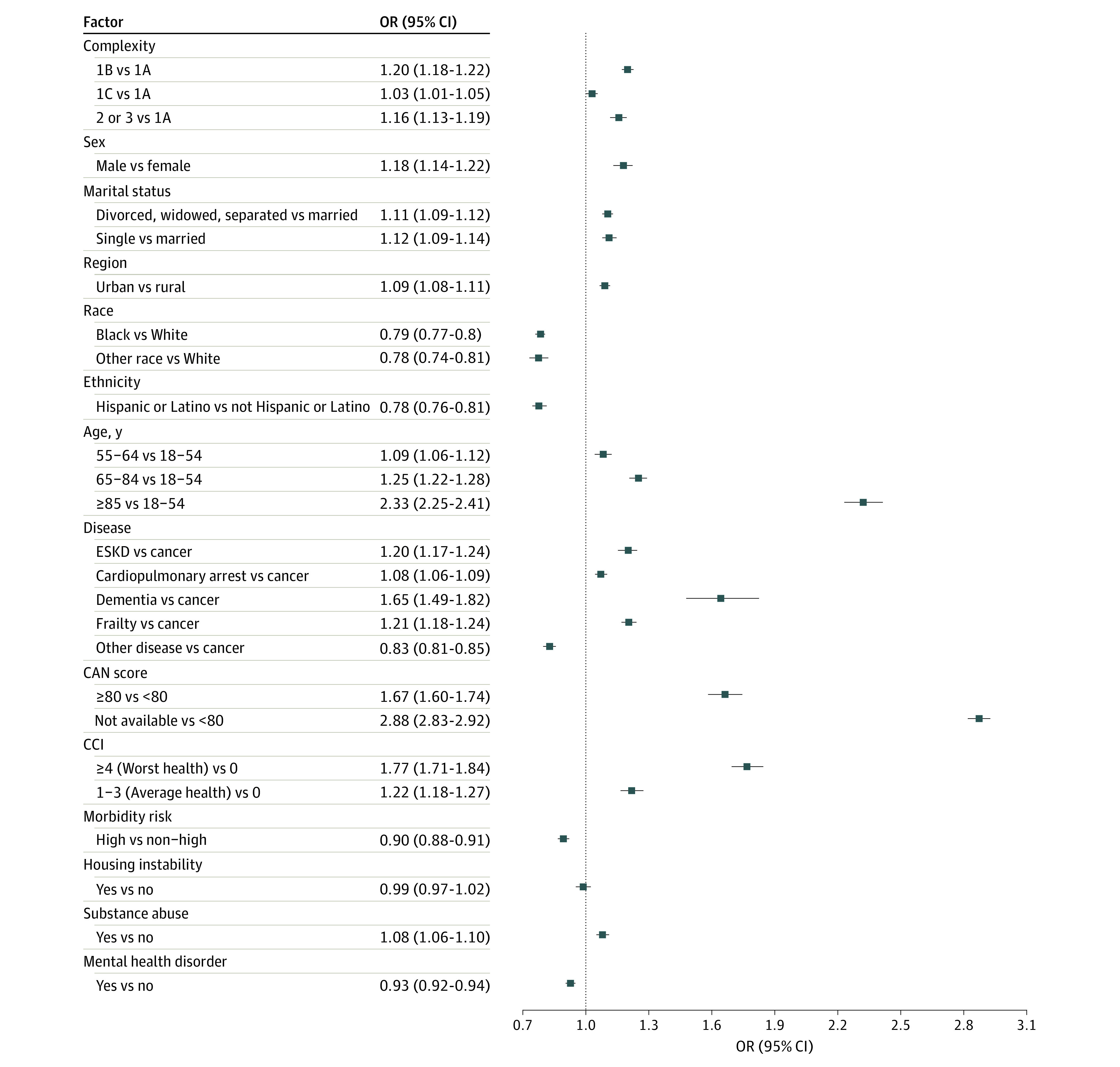
Odds Ratios (ORs) of Select Factors on Preoperative Life-Sustaining Treatment Documentation for Veterans CAN indicates care assessment need score; CCI, Charlson Comorbidity Index; ESKD, end-stage kidney disease.

Figure 2 shows the estimates from the covariate-adjusted regression model. The odds of completing preoperative LST were lower for Black veterans (OR, 0.79; 95% CI, 0.77-0.80; *P* < .001), other veterans (OR, 0.78; 95% CI, 0.74-0.81; *P* < .001), compared with White individuals (eTable in Supplement 1). Rurality did not modify the association between patient’s race and preoperative LST completion. Hispanic patients were less likely to complete LST than non-Hispanic patients (OR, 0.78; 95% CI, 0.76-0.81; *P* < .001) (eTable in Supplement 1). Patients with mental health disorder history also had lower likelihood (OR, 0.93; 95% CI, 0.92-0.94; *P* = .001) of completing an LST note before surgery than those without such history. Male patients (OR, 1.18; 95% CI, 1.14-1.22; *P* < .001), those with history of substance use (OR, 1.08; 95% CI, 1.06-1.10; *P* = .004), and those who resided in urban areas (OR, 1.09; 95% CI, 1.08-1.11; *P* < .001) were more likely to complete preoperative LST notes compared with their counterparts. Patients who underwent surgery at level 1A facilities, which have the highest complexity, had the least likelihood of completing preoperative LST notes. After we performed a sensitivity analysis on the logistic regression results with a wider time window for preoperative LST completion, there was minimal change observed in the findings. Odds ratios associated with factors with increased likelihood of preoperative LST documentation exhibited a greater magnitude. The overall conclusion and interpretation drawn from the study remained unaffected.

With each passing year, patients undergoing surgical procedures had greater likelihoods of completing LST before surgery. Given the potential impact of the COVID-19 pandemic on LST completion rates, we examined the number of completed mechanical ventilation questions in the LST note over time. However, no significant association was found.

## Discussion

We assessed disparities in preoperative GOC documentation for patients undergoing surgical procedures within the VHA system. We identified important risks for poor GOC documentation, including novel associations with patients’ history of mental health disorder. Despite increasing rates of preoperative LST documentation over time, even during the COVID-19 pandemic, a low proportion of patients undergoing surgical procedures overall, particularly individuals from racially and ethnically minoritized communities and patients from rural regions, completed LST documentation before surgery. While there were greater odds of clinicians completing preoperative LST documentation for patients from racially or ethnically minoritized communities at lower-complexity VA facilities, patients undergoing surgical procedures from racially or ethnically minoritized communities, patients with a history of comorbid mental health disorders, and those who live in rural areas were significantly less likely to complete LST documentation.

Our study provides several opportunities for health care clinicians to engage with individuals from racially and ethnically minoritized communities in GOC discussions and improve preoperative goals documentation. Preoperative LST documentation rates were significantly lower for Black patients, Hispanic patients, and patients identifying as other (ie, American Indian or Alaskan Native, Asian, Native Hawaiian, or Other Pacific Islander) than for White patients. Although our study focuses on patients undergoing surgical procedures, racial and ethnic disparities in GOC planning have been reported in other studies.^20,21,22^ Rurality of residence also suggests cultural differences on an individual basis. Previous studies^6,23^ have shown differences in LST documentation rates between urban and rural residents. However, in our study, rurality did not compound racial disparities in preoperative LST documentation. Elderly patients aged 65 years and older also tended to complete preoperative LST documentation, in line with a prior VA-based study^23^ that found that patients who were older and seriously ill were more likely to have GOC documentation. Yet, patients who are not elderly can also have serious illnesses and derive benefit from GOC planning.^24,25^

Patients undergoing surgical procedures with comorbid psychiatric disability are also at risk for not engaging in preoperative GOC discussions. One in 5 US adults live with a mental health condition, and about 1 in 20 live with serious psychiatric disability.^26^ A cross-sectional study of psychiatric inpatients determined that 60% of the patients exhibited incapacity to make medical decisions about their care, underscoring the difficulties patients with comorbid mental health disabilities may face before a stressful and significant event like surgery.^27^ Patients with serious mental disabilities express interest in advance care planning, yet few have discussed their preferences with clinicians.^28^

Our study’s novel finding that a history of mental health disabilities was associated with lower rates of LST documentation highlights potential harmful biases clinicians may hold about these patients. One study^29^ found that participants described feelings that clinicians do not treat people with mental health disabilities in a holistic manner; poor patient-clinician communication could extend to GOC discussions. For patients with serious mental disabilities, identifying surrogates for decision-making in cases where patient capacity is compromised is also important as social, legal, and ethical challenges may arise with conservators, illustrated by the lawsuit Conservatorship of Wendland.^30^ The patient was conscious but functionally impaired from a devastating accident; without an advance directive designating a durable power of attorney, his wife was his conservator, who was privy to knowledge that he did not want to live in a persistently vegetative state. However, when she, supported by the hospital ethics committee, elected to withdraw artificial hydration and nutrition, the patient’s other relatives objected to the withdrawal of LSTs, resulting in litigation. In his analysis of that case, West^30^ argues that clear patient preferences should be documented and, if not, difficult medical decisions be put before a court for arbitration.

We found an encouraging positive trend in preoperative LST documentation, despite the COVID-19 pandemic impacting overall care delivery for health systems nationwide. Indeed, patients undergoing surgery each subsequent year had higher likelihoods of completing LST documentation prior to surgery, compared with the initial rollout of the LST program.^12^ Although our data cannot address these possibilities, this phenomenon suggests effective LSTDI implementation within the VHA system, though other factors to consider are that total surgery numbers did decrease due to policies enacted during the pandemic or that the procedures that occurred may be for patients who are more sick and especially needed intervention. The proportions of high-risk or non–high-risk procedures remained comparable over time, despite the onset of the pandemic. However, we were unable to examine how individual clinicians and hospitals determined which surgical procedures to proceed during COVID-19. While the proportion of completed preoperative LST notes was nearly one-fifth of the total surgical population by the end of the study period, additional efforts to implement LST communication and documentation are still needed.

### Limitations

This study has limitations. The main outcome is LST documentation, even though it is possible that GOC discussions occurred but with poor documentation. However, documentation is a core aspect of communication for care planning, and clinicians cannot act on patients’ expressed preferences for future health care without it. We investigated a 30-day time window prior to surgery for preoperative LST documentation; however, sensitivity analyses for a 90-day period produced comparable results in LST completion. Our model also could not account for all possible factors, including the diagnostic vs therapeutic nature of procedures. Our study relies on a cohort drawn from the VHA surgical patient population, which may potentially limit the generalizability of our findings. Nonetheless, the VHA system is exemplary of highly integrated palliative care services, and the LST program may serve as a model for other hospitals to increase rates of GOC discussions. We recognize that the database we investigated does not include all possible patient-level, clinician-level, or system-level factors that could impact disparities in LST completion, and future studies should incorporate elements from health inequities frameworks to further characterize gaps in and opportunities to improve GOC planning for patients undergoing surgical procedures.

## Conclusions

Among veterans who underwent surgery, patients from racially and ethnically minoritized communities, rural residents, and those who had a history of comorbid mental health disabilities completed preoperative LST documentation less frequently, despite national policy implementation. While patients presenting to VA facilities with lower facility-complexity levels completed preoperative LST less often, additional studies incorporating regional and system-level variabilities are necessary to understand LST completion within the hospital setting, especially for the surgical patient population. Our study reinforces the persistence of racial and ethnic disparities and highlights other patient-level gaps in GOC documentation for the surgical patient population. Our study also supports the need for future interventions that target patient groups at risk of not engaging in serious illness communication.

## Notes

Supplement 1. **eTable.** Adjusted Analysis of Patient- and System-Level Characteristics With Preoperative Life-Sustaining Treatment (LST) Documentation

Supplement 2.
Data Sharing Statement
