# Supplementary material for: Disparities in Preoperative Goals of Care Documentation in Veterans
Source: JAMA Netw Open. 2023 Dec 19;6(12):e2348235. doi: 10.1001/jamanetworkopen.2023.48235 (PMC10731481; doi:10.1001/jamanetworkopen.2023.48235)
Supplement: Supplement 1. — eTable. Adjusted Analysis of Patient- and System-Level Characteristics With Preoperative Life-Sustaining Treatment (LST) Documentation [file jamanetwopen-e2348235-s001.pdf]

Supplemental Online Content

Wu A, Giannitrapani KF, Garcia A, et al. Disparities in preoperative goals of care documentation in veterans. *JAMA Netw Open*. 2023;6(12):e2348235.
doi:10.1001/jamanetworkopen.2023.48235

eTable. Adjusted Analysis of Patient- and System-Level Characteristics With Preoperative Life-Sustaining Treatment (LST) Documentation

This supplemental material has been provided by the authors to give readers additional information about their work.

© 2023 Wu A et al. *JAMA Network Open.*

eTable 1: Adjusted Analysis of Patient- and System-level Characteristics with Preoperative Life-Sustaining Treatment (LST) Documentation		
	Odds Ratio (95% Confidence Interval) (Ref = no preoperative LST completed)	P value
**Age Group**		
18-54	Ref	
55-64	1.086 (1.056-1.116)	0.043
65-84	1.252 (1.220-1.284)	<0.001
85+	2.326 (2.245-2.409)	<0.001
**Gender**		
Female	Ref	
Male	1.180 (1.144-1.217)	<0.001
**Race**		
White	Ref	
Black	0.785 (0.772-0.799)	<0.001
Other	0.778 (0.743-0.814)	<0.001
**Ethnicity**		
Non-Hispanic	Ref	
Hispanic	0.783 (0.759-0.807)	<0.001
**Marital Status**		
Married	Ref	
Divorced/widowed/separated	1.107 (1.092-1.122)	<0.001
Single/never married	1.115 (1.092-1.140)	0.001
**Rurality**		
Urban	Ref	
Rural	0.914 (0.928-0.902)	<0.001
**CAN score (1 year)**		
<80	Ref	
≥80	1.665 (1.595-1.739)	<0.001

© 2023 Wu A et al. *JAMA Network Open.*

Not available	2.877 (2.833-2.921)	<0.001
**Charlson Comorbidity Index**		
0 (Lowest comorbidity level)	Ref	
1-3	1.223 (1.180-1.267)	<0.001
4+ (High comorbidity level)	1.772 (1.709-1.837)	<0.001
**Co-morbid Disease**		
Cancer	Ref	
ESRD	1.202 (1.166-1.238)	<0.001
Cardiopulmonary Disease	1.075 (1.056-1.095)	0.007
Dementia	1.646 (1.491-1.819)	0.001
Frailty	1.209 (1.183-1.235)	<0.001
Other	0.829 (0.810-0.849)	<0.001
Not available	0.822 (0.789-0.856)	0.001
**Substance Use**		
None	Ref	
Yes (1 or more)	1.082 (1.063-1.102)	0.004
**Mental Health Co-morbidity**		
None	Ref	
Yes (1 or more)	0.93 (0.917-0.944)	0.001
**Housing Instability**		
None	Ref	
Yes	0.991 (0.966-1.016)	0.806
**Surgical Specialty**		
General	Ref	
Neurosurgery	0.821 (0.799-0.844)	<0.001
Cardiothoracic	1.204 (1.178-1.229)	<0.001
Vascular	0.736 (0.715-0.758)	<0.001
Orthopedic	1.048 (1.030-1.066)	0.066

© 2023 Wu A et al. *JAMA Network Open.*

Urology	0.411 (0.393-0.430)	<0.001
Other	0.897 (0.856-0.940)	0.119
**Surgical Risk**		
Non-high risk	Ref	
High-risk	0.896 (0.879-0.914)	<0.001
**Procedure Year**		
2017	Ref	
2018	15.831 (15.015-16.693)	<0.001
2019	27.174 (25.782-28.641)	<0.001
2020	46.976 (44.365-49.361)	<0.001
2021	61.221 (57.992-64.630)	<0.001
2022	67.375 (63.737-71.221)	<0.001
**VA Facility Complexity**		
1a	Ref	
1b	1.201 (1.183-1.220)	<0.001
1c	1.031 (1.011-1.052)	0.294
2 or 3	1.158 (1.129-1.188)	<0.001

© 2023 Wu A et al. *JAMA Network Open.*
