# Supplementary material for: Disparities in Preoperative Goals of Care Documentation in Veterans
Source: JAMA Netw Open. 2023 Dec 19;6(12):e2348235. doi: 10.1001/jamanetworkopen.2023.48235 (PMC10731481; doi:10.1001/jamanetworkopen.2023.48235)
Supplement: Supplement 2. — Data Sharing Statement [file jamanetwopen-e2348235-s002.pdf]

Data Sharing Statement

Wu. Disparities in Preoperative Goals of Care Documentation in Veterans. *JAMA Netw Open*. Published December 19, 2023. doi:10.1001/jamanetworkopen.2023.48235

Data
Data available: No

Additional Information
Explanation for why data not available: All data is available through the VA Corporate Data Warehouse and can be accessed with appropriate VA permissions. The authors will not share data.
